# Supplementary material for: Prevalence and correlates of compliance with 24-h movement guidelines among children from urban and rural Kenya—The Kenya-LINX project
Source: PLoS One. 2022 Dec 30;17(12):e0279751. doi: 10.1371/journal.pone.0279751 (PMC9803245; doi:10.1371/journal.pone.0279751)
Supplement: S1 Table — (DOCX) [file pone.0279751.s001.docx]

| **Table S1 (supplementary material)** | | | | | | | |
| --- | --- | --- | --- | --- | --- | --- | --- |
| **Potential correlates of meeting movement guidelines** | | | | | | | |
| **Child level correlates** | **Urban** | | **Rural** | | **t-value/chi-squared** | **P value** | **Use in the analysis** |
| **Age (years)** | 10.9 | 0.74 | 11.3 | 0.95 | 4.15 | <0.01 | Continuous |
| **Sex (% female)** | 52.4 |  | 54.0 |  | 0.17 | 0.68 | Dichotomous |
| **Weight status (IOTF)** |  |  |  |  | 55.22 | <0.01 | Re-coded to 3 levels:  i) below healthy weight,  ii) healthy weight  iii) above a healthy weight |
| **Thinness grade 3 (%)** | 2.5 |  | 3.4 |  |  | 0.99 |  |
| **Thinness grade 2 (%)** | 1.8 |  | 15.3 |  |  | <0.01 |  |
| **Thinness grade 1 (%)** | 11.2 |  | 22.6 |  |  | 0.01 |  |
| **Normal weight (%)** | 63.2 |  | 51.4 |  |  | 0.17 |  |
| **Overweight (%)** | 16.6 |  | 6.21 |  |  | 0.01 |  |
| **Obese (%)** | 4.7 |  | 0.50 |  |  | 0.16 |  |
| **Screen time before school (% yes)** | 55.3 |  | 39.91 |  | 10.72 | <0.01 | Dichotomous |
| **Exercise before school (% yes)** | 64.8 |  | 62.22 |  | 0.32 | 0.57 |  |
| **Homework before school (% yes)** | 87.3 |  | 68.13 |  | 25.84 | <0.01 |  |
| **Active transport to school (yes)** | 57.7 |  | 66.52 |  | 3.64 | 0.06 |  |
| **Active transport from school (% yes)** | 64.8 |  | 66.51 |  | 0.14 | 0.70 |  |
| **Breaktime** |  |  |  |  | 35.10 | <0.01 | Categorical |
| *Sat around* | 47.89 |  | 26.13 |  |  | <0.01 |  |
| *Ran around* | 34.51 |  | 61.11 |  |  | <0.01 |  |
| *Stood around* | 2.11 |  | 3.19 |  |  | 1.00 |  |
| *Walked around* | 15.49 |  | 9.57 |  |  | 1.00 |  |
| **Lunch time** |  |  |  |  | 8.62 | 0.035 | Categorical |
| *Sat around* | 36.27 |  | 30.85 |  |  | 1.00 |  |
| *Ran around* | 41.55 |  | 54.25 |  |  | 0.05 |  |
| *Stood around* | 4.58 |  | 4.25 |  |  | 1.00 |  |
| *Walked around* | 17.61 |  | 10.64 |  |  | 0.30 |  |
| **After school screen** | 75.00 |  | 65.42 |  | 5.06 | 0.03 | Dichotomous |
| **After school exercise** | 75.70 |  | 77.11 |  | 0.23 | 0.72 |  |
| **After school homework** | 94.72 |  | 71.27 |  | 49.8 | <0.01 |  |
| **Can you ride a bike?** | 84.86 |  | 76.10 |  | 5.77 | 0.02 |  |
| **Can you swim?** | 76.76 |  | 69.68 |  | 2.94 | 0.08 |  |
| **Sport club out of school** | 84.86 |  | 84.57 |  | 0.01 | 0.93 |  |
| **Sport club in school** | 85.21 |  | 88.30 |  | 0.92 | 0.24 |  |
| **Brownies, Guides or Scouts** | 45.77 |  | 35.64 |  | 4.78 | 0.03 |  |
| **Do your parents encourage you to be physically active** | 91.20 |  | 89.89 |  | 0.23 | 0.63 |  |
| **School level correlates** | | | | | | | |
| **Duration of the school day (hours)** | 9.03 (1.01) |  | 8.56 (0.75 |  | -5.11 | <0.01 | Continuous |
| **School transport for extracurricular activities** | 52.30 |  | 37.27 |  | 8.75 | <0.01 | Dichotomous |
| **School health committee (yes)** | 83.26 |  | 51.55 |  | 46.51 | <0.01 | Dichotomous |
| **Short breaks < 30 minutes (one)** |  |  |  |  |  |  |  |
| *Two* | 52.72 |  | 80.75 |  | 32.79 | <0.01 | Dichotomous |
| **Long breaks > 30 minutes** |  |  |  |  | 58.27 | <0.01 |  |
| *one* | 33.47 |  | 15.53 |  |  | <0.01 | Categorical |
| *Two* | 26.78 |  | 65.22 |  |  | <0.01 |  |
| *Three* | 39.75 |  | 19.25 |  |  | <0.01 |  |
| **PE sessions per week** | 3.69 (1.12) |  | 4.81 (0.4) |  | 14.07 | <0.01 | Continuous |
| **Duration of PE sessions** |  |  |  |  | 160.41 | <0.01 | Categorical |
| *Short <35 minutes (%)* | 49.37 |  | 0.00 |  |  | <0.01 |  |
| *Medium 35-45 minutes (%)* | 50.63 |  | 68.32 |  |  | <0.01 |  |
| *Long 46-60 minutes (%)* | 0.00 |  | 31.68 |  |  | <0.01 |  |
