# Supplementary material for: Prevalence and correlates of compliance with 24-h movement guidelines among children from urban and rural Kenya—The Kenya-LINX project
Source: PLoS One. 2022 Dec 30;17(12):e0279751. doi: 10.1371/journal.pone.0279751 (PMC9803245; doi:10.1371/journal.pone.0279751)
Supplement: S2 Table — (DOCX) [file pone.0279751.s002.docx]

| **Table S2 (supplementary material)** | | | | | | | | | | | | |
| --- | --- | --- | --- | --- | --- | --- | --- | --- | --- | --- | --- | --- |
| **Univariate analysis of correlates of meeting the combined movement guidelines** | | | | | | | | | | | | |
|  | **PA** | | | | **Screen** | | | | **Sleep** | | | |
|  | **OR** | **95% CI** | | **p-value** | **OR** | **95% CI** | | **p-value** | **OR** | **95% CI** | | **p-value** |
| **County (Nairobi)** | **0.49** | **0.33** | **0.73** | **0.00** | 0.83 | 0.57 | 1.20 | 0.32 | **0.45** | **0.29** | **0.71** | **0.00** |
| **School (Public)** | **1.42** | **0.95** | **2.10** | **0.08** | 1.04 | 0.71 | 1.51 | 0.85 | **0.56** | **0.34** | **0.91** | **0.02** |
| **Sex (Girl)** | **3.47** | **2.30** | **5.24** | **0.00** | 0.74 | 0.52 | 1.07 | 0.12 | 0.88 | 0.56 | 1.38 | 0.58 |
| **Age (years)** | 0.94 | 0.75 | 1.18 | 0.60 | 1.12 | 0.90 | 1.40 | 0.30 | **0.62** | **0.46** | **0.82** | **0.00** |
| **Weight status (Healthy weight)** |  |  |  |  |  |  |  |  |  |  |  |  |
| *Below a healthy weight* | 1.33 | 0.81 | 2.18 | 0.26 | 1.18 | 0.76 | 1.84 | 0.46 | 1.09 | 0.64 | 1.86 | 0.75 |
| *Above a healthy weight* | **0.32** | **0.19** | **0.55** | **0.00** | 1.42 | 0.83 | 2.44 | 0.20 | 0.90 | 0.46 | 1.76 | 0.76 |
| **Screen time before school (No)** | **1.24** | **0.98** | **1.57** | **0.07** | **2.03** | **1.40** | **2.95** | **0.00** | 1.01 | 0.64 | 1.59 | 0.96 |
| **Exercise before school** | 0.86 | 0.58 | 1.27 | 0.45 | 1.17 | 0.80 | 1.71 | 0.43 | 1.12 | 0.70 | 1.78 | 0.64 |
| **Homework before school** | **1.78** | **1.06** | **2.97** | **0.03** | 1.35 | 0.85 | 2.14 | 0.21 | 0.91 | 0.51 | 1.61 | 0.75 |
| **Active transport to school (yes)** | **1.53** | **1.04** | **2.26** | **0.03** | **1.60** | **1.10** | **2.33** | **0.01** | 1.45 | 0.90 | 2.34 | 0.13 |
| **Active transport from school** | **1.42** | **0.95** | **2.11** | **0.08** | 0.94 | 0.64 | 1.38 | 0.74 | **1.59** | **0.96** | **2.62** | **0.07** |
| **Breaktime (sat around)** |  |  |  |  |  |  |  |  |  |  |  |  |
| *Ran around* | **2.47** | **1.61** | **3.80** | **0.00** | 1.06 | 0.71 | 1.57 | 0.79 | **1.77** | **1.06** | **2.94** | **0.03** |
| *Stood around* | **8.57** | **1.08** | **67.68** | **0.04** | 2.19 | 0.57 | 8.34 | 0.25 | 1.12 | 0.23 | 5.39 | 0.89 |
| *Walked around* | 1.15 | 0.64 | 2.07 | 0.63 | 0.73 | 0.41 | 1.30 | 0.28 | 1.49 | 0.72 | 3.09 | 0.29 |
| **Lunch time (Sat around)** |  |  |  |  |  |  |  |  |  |  |  |  |
| *Ran around* | **2.31** | **1.49** | **3.57** | **0.00** | 0.96 | 0.64 | 1.45 | 0.85 | 1.20 | 0.73 | 1.99 | 0.48 |
| *Stood around* | 1.54 | 0.59 | 4.02 | 0.38 | 0.80 | 0.32 | 2.00 | 0.64 | 0.70 | 0.19 | 2.52 | 0.58 |
| *Walked around* | 1.30 | 0.73 | 2.32 | 0.37 | 1.24 | 0.69 | 2.20 | 0.47 | 0.78 | 0.37 | 1.66 | 0.52 |
| **After school screen** | 0.93 | 0.61 | 1.42 | 0.75 | **3.78** | **2.38** | **6.00** | **0.00** | 0.93 | 0.56 | 1.54 | 0.78 |
| **After school exercise** | **0.56** | **0.36** | **0.86** | **0.01** | **1.75** | **1.12** | **2.73** | **0.01** | 0.66 | 0.37 | 1.17 | 0.15 |
| **After school homework** | **1.80** | **0.99** | **3.27** | **0.05** | **2.76** | **1.52** | **4.98** | **0.00** | **1.66** | **0.93** | **2.98** | **0.09** |
| **Can you ride a bike** | 1.40 | 0.87 | 2.26 | 0.17 | **0.55** | **0.33** | **0.90** | **0.02** | 1.26 | 0.69 | 2.32 | 0.46 |
| **Can you swim** | 1.33 | 0.86 | 2.03 | 0.20 | 0.84 | 0.55 | 1.28 | 0.41 | **1.77** | **1.00** | **3.12** | **0.05** |
| **Sport club out of school** | 0.76 | 0.45 | 1.27 | 0.29 | **1.67** | **0.98** | **2.85** | **0.06** | 0.61 | 0.30 | 1.23 | 0.17 |
| **Sport club in school** | **0.52** | **0.30** | **0.88** | **0.02** | **2.44** | **1.34** | **4.44** | **0.00** | 0.53 | 0.25 | 1.16 | 0.11 |
| **clubs or guides** | 1.21 | 0.82 | 1.80 | 0.33 | **0.59** | **0.40** | **0.85** | **0.00** | 1.44 | 0.92 | 2.27 | 0.11 |
| **Do your parents encourage you to be PA** | **1.75** | **0.94** | **3.28** | **0.08** | 0.69 | 0.36 | 1.32 | 0.26 | 0.96 | 0.45 | 2.08 | 0.93 |
| **Duration of the school day** | **1.24** | **0.98** | **1.57** | **0.07** | 0.93 | 0.75 | 1.15 | 0.49 | 1.14 | 0.87 | 1.50 | 0.33 |
| **Transport for extracurricular activities** | **0.48** | **0.31** | **0.74** | **0.00** | 1.06 | 0.71 | 1.59 | 0.78 | 0.96 | 0.58 | 1.58 | 0.86 |
| **School healthy committee (yes)** | **0.66** | **0.41** | **1.06** | **0.09** | 1.04 | 0.69 | 1.58 | 0.85 | **2.01** | **1.13** | **3.56** | **0.02** |
| **Short breaks (one)** |  |  |  |  |  |  |  |  |  |  |  |  |
| *Two* | **1.75** | **0.94** | **3.28** | **0.08** | 0.93 | 0.75 | 1.15 | 0.49 | 1.14 | 0.87 | 1.50 | 0.33 |
| **Long breaks (1)** |  |  |  |  |  |  |  |  |  |  |  |  |
| *Two* | **2.12** | **1.25** | **3.61** | **0.01** | 0.67 | 0.40 | 1.14 | 0.14 | 1.29 | 0.72 | 2.31 | 0.40 |
| *Three* | **1.87** | **1.52** | **2.29** | **0.00** | 1.03 | 0.85 | 1.24 | 0.76 | **0.33** | **0.15** | **0.72** | **0.01** |
| **PE sessions per week** | **1.87** | **1.52** | **2.29** | **0.00** | 1.03 | 0.85 | 1.24 | 0.76 | **1.68** | **1.25** | **2.26** | **0.00** |
| **Duration PE (short)** |  |  |  |  |  |  |  |  |  |  |  |  |
| *Medium* | **4.06** | **2.53** | **6.52** | **0.00** | 1.00 | 0.64 | 1.57 | 0.98 | **3.38** | **1.65** | **6.90** | **0.00** |
| *Long* | **5.92** | **2.64** | **13.27** | **0.00** | 1.31 | 0.66 | 2.60 | 0.44 | **2.63** | **1.02** | **6.79** | **0.05** |

| **Table S2 continued (supplementary material)** | | | | | | | | | | | | | |
| --- | --- | --- | --- | --- | --- | --- | --- | --- | --- | --- | --- | --- | --- |
| **Univariable analysis of correlates of meeting the combined movement guidelines** | | | | | | | | | | | | | |
|  | **PA + Screen** | | | | **PA + Sleep** | | | | **Sleep + Screen** | | | | |
|  | **OR** | **95% CI** | | **p-value** | **OR** | **95% CI** | | **p-value** | **OR** | **95% CI** | | **p-value** |  |
| **County (Nairobi)** | **0.68** | **0.46** | **1.01** | **0.06** | **0.54** | **0.26** | **1.09** | **0.09** | 0.75 | 0.27 | 2.10 | 0.58 |  |
| **School (Public)** | **1.40** | **0.95** | **2.08** | **0.09** | **0.40** | **0.17** | **0.94** | **0.04** | 1.04 | 0.36 | 2.96 | 0.95 |  |
| **Sex (Girl)** | **1.66** | **1.12** | **2.46** | **0.01** | **2.10** | **1.01** | **4.37** | **0.05** | 0.08 | **0.01** | **0.59** | **0.01** |  |
| **Age (years)** | 1.16 | 0.92 | 1.46 | 0.22 | **0.67** | **0.43** | **1.05** | **0.08** | 1.02 | 0.55 | 1.87 | 0.95 |  |
| **Weight status (Healthy weight)** |  |  |  |  |  |  |  |  |  |  |  |  |  |
| *Below a healthy weight* | 1.35 | 0.85 | 2.14 | 0.20 | 0.45 | 0.17 | 1.21 | 0.11 | 2.34 | 0.74 | 7.42 | 0.15 |  |
| *Above a healthy weight* | 0.68 | 0.37 | 1.25 | 0.21 | 0.44 | 0.13 | 1.52 | 0.20 | 1.26 | 0.25 | 6.36 | 0.78 |  |
| **Screen time before school (No)** | **1.47** | **0.99** | **2.18** | **0.05** | 0.69 | 0.34 | 1.42 | 0.32 |  |  |  |  |  |
| **Exercise before school** | 0.82 | 0.54 | 1.23 | 0.34 | 1.16 | 0.56 | 2.39 | 0.70 | 2.06 | 0.73 | 5.79 | 0.17 |  |
| **Homework before school** | 1.41 | 0.89 | 2.26 | 0.15 | 0.86 | 0.35 | 2.15 | 0.75 | 0.98 | 0.27 | 3.54 | 0.97 |  |
| **Active transport to school (yes)** | **1.42** | **0.95** | **2.14** | **0.09** | 0.74 | 0.37 | 1.52 | 0.42 | 1.77 | 0.56 | 5.65 | 0.33 |  |
| **Active transport from school** | 1.15 | 0.76 | 1.73 | 0.52 | 0.92 | 0.44 | 1.92 | 0.82 | 2.15 | 0.60 | 7.75 | 0.24 |  |
| **Breaktime (sat around)** |  |  |  |  |  |  |  |  |  |  |  |  |  |
| *Ran around* | 1.27 | 0.83 | 1.95 | 0.27 | 1.71 | 0.78 | 3.79 | **0.18** | **2.99** | **0.81** | **11.03** | **0.10** |  |
| *Stood around* | **3.49** | **1.06** | **11.47** | **0.04** | 1.59 | 0.19 | 13.58 | 0.67 | 0.00 | 0.00 | Inf | 0.99 |  |
| *Walked around* | 0.87 | 0.45 | 1.66 | 0.67 | 0.89 | 0.24 | 3.34 | 0.86 | 2.02 | 0.33 | 12.39 | 0.45 |  |
| **Lunch time (Sat around)** |  |  |  |  |  |  |  |  |  |  |  |  |  |
| *Ran around* | **1.56** | **0.99** | **2.46** | **0.05** | 1.59 | 0.73 | 3.48 | 0.24 | 1.10 | 0.31 | 3.97 | 0.88 |  |
| *Stood around* | **2.75** | **1.09** | **6.96** | **0.03** | 0.76 | 0.09 | 6.21 | 0.79 | 1.96 | 0.21 | 18.44 | 0.56 |  |
| *Walked around* | 1.48 | 0.80 | 2.73 | 0.21 | 0.22 | 0.03 | 1.74 | 0.15 | 2.38 | 0.58 | 9.80 | 0.23 |  |
| **After school screen** | **1.86** | **1.22** | **2.82** | **0.00** | **0.42** | **0.16** | **1.11** | **0.08** | 1.68 | 0.59 | 4.81 | 0.34 |  |
| **After school exercise** | 1.05 | 0.67 | 1.65 | 0.84 | **0.30** | **0.09** | **1.01** | **0.05** | **3.88** | **1.37** | **10.95** | **0.01** |  |
| **After school homework** | **1.86** | **1.10** | **3.13** | **0.02** | 0.57 | 0.17 | 1.91 | 0.36 | **3.07** | **1.02** | **9.28** | **0.05** |  |
| **Can you ride a bike** | **0.67** | **0.41** | **1.09** | **0.10** | **3.78** | **0.89** | **16.09** | **0.07** | 0.44 | 0.15 | 1.33 | 0.15 |  |
| **Can you swim** | 0.93 | 0.60 | 1.44 | 0.75 | 1.64 | 0.66 | 4.06 | 0.29 | 0.97 | 0.30 | 3.10 | 0.96 |  |
| **Sport club out of school** | 1.29 | 0.76 | 2.18 | 0.35 | 0.99 | 0.37 | 2.66 | 0.99 | 0.85 | 0.19 | 3.85 | 0.83 |  |
| **Sport club in school** | 1.37 | 0.79 | 2.38 | 0.26 | 0.62 | 0.18 | 2.09 | 0.44 | 0.98 | 0.22 | 4.45 | 0.98 |  |
| **clubs or guides** | **0.67** | **0.45** | **1.00** | **0.05** | **2.28** | **1.10** | **4.70** | **0.03** | 0.93 | 0.33 | 2.65 | 0.89 |  |
| **Do your parents encourage you to be PA** | 0.87 | 0.45 | 1.68 | 0.68 | 0.73 | 0.24 | 2.17 | 0.57 | 1.45 | 0.19 | 11.33 | 0.72 |  |
| **Duration of the school day** | 1.15 | 0.91 | 1.44 | 0.24 | 1.25 | 0.84 | 1.87 | 0.27 | 0.67 | 0.30 | 1.53 | 0.34 |  |
| **offer school transport** | **0.57** | **0.37** | **0.88** | **0.01** | 0.74 | 0.34 | 1.61 | 0.44 | 1.46 | 0.44 | 4.86 | 0.54 |  |
| **School healthy committee (yes)** | 1.21 | 0.77 | 1.88 | 0.41 | **0.39** | **0.18** | **0.84** | **0.02** | 0.72 | 0.21 | 2.51 | 0.61 |  |
| **Short breaks (one)** |  |  |  |  |  |  |  |  |  |  |  |  |  |
| *Two* | 1.21 | 0.77 | 1.88 | 0.41 | **2.16** | **0.86** | **5.46** | **0.10** | 2.61 | 0.56 | 12.27 | 0.22 |  |
| **Long breaks (1)** |  |  |  |  |  |  |  |  |  |  |  |  |  |
| *Two* | **1.85** | **1.07** | **3.21** | **0.03** | **2.21** | **0.86** | **5.71** | **0.10** | **0.15** | **0.03** | **0.72** | **0.02** |  |
| *Three* | 1.49 | 0.83 | 2.67 | 0.18 | **0.27** | **0.05** | **1.35** | **0.11** | **0.10** | **0.01** | **0.80** | **0.03** |  |
| **PE sessions per week** | **1.21** | **0.98** | **1.49** | **0.07** | **1.44** | **0.93** | **2.22** | **0.10** | 1.62 | 0.78 | 3.37 | 0.20 |  |
| **Duration PE (short)** |  |  |  |  |  |  |  |  |  |  |  |  |  |
| *Medium* | 1.50 | 0.91 | 2.46 | 0.11 | **2.85** | **0.96** | **8.50** | **0.06** | 4.88 | 0.61 | 38.95 | 0.14 |  |
| *Long* | 1.74 | 0.86 | 3.52 | 0.12 | **1.78** | **0.38** | **8.26** | **0.46** | 2.44 | 0.15 | 39.77 | 0.53 |  |

| **Table S2 continued (supplementary material)** | | | | |
| --- | --- | --- | --- | --- |
| **Univariate analysis of correlates of meeting the combined movement guidelines** | | | | |
|  | **Sleep + Screen** | | | |
|  | **OR** | **95% CI** | | **p-value** |
| **County (Nairobi)** | **0.44** | **0.22** | **0.90** | **0.02** |
| **School (Public)** | 0.73 | 0.35 | 1.54 | 0.41 |
| **Sex (Girl)** | 0.89 | 0.44 | 1.80 | 0.75 |
| **Age (years)** | **0.64** | **0.41** | **1.00** | **0.05** |
| **Weight status (Healthy weight)** |  |  |  |  |
| *Below a healthy weight* | 1.57 | 0.73 | 3.38 | 0.24 |
| *Above a healthy weight* | 0.61 | 0.17 | 2.12 | 0.44 |
| **Screen time before school (No)** | **3.40** | **1.51** | **7.68** | **0.00** |
| **Exercise before school** | 0.96 | 0.46 | 1.99 | 0.91 |
| **Homework before school** | 1.45 | 0.65 | 3.22 | 0.36 |
| **Active transport to school (yes)** | **2.59** | **1.10** | **6.08** | **0.03** |
| **Active transport from school** | 1.51 | 0.69 | 3.31 | 0.31 |
| **Breaktime (sat around)** |  |  |  |  |
| *Ran around* | **2.93** | **1.22** | **7.02** | **0.02** |
| *Stood around* | 2.31 | 0.26 | 20.49 | 0.45 |
| *Walked around* | 1.75 | 0.50 | 6.21 | 0.38 |
| **Lunch time (Sat around)** |  |  |  |  |
| *Ran around* | 1.51 | 0.69 | 3.32 | 0.31 |
| *Stood around* | - | - | - | - |
| *Walked around* | 0.92 | 0.28 | 3.02 | 0.88 |
| **After school screen** | **1.81** | **0.89** | **3.70** | **0.10** |
| **After school exercise** | 0.53 | 0.20 | 1.41 | 0.21 |
| **After school homework** | **2.68** | **1.22** | **5.88** | **0.01** |
| **Can you ride a bike** | 1.36 | 0.51 | 3.61 | 0.54 |
| **Can you swim** | **2.80** | **0.97** | **8.11** | **0.06** |
| **Sport club out of school** | 0.33 | 0.08 | 1.40 | 0.13 |
| **Sport club in school** | 0.38 | 0.09 | 1.62 | 0.19 |
| **clubs or guides** | 1.11 | 0.55 | 2.24 | 0.77 |
| **Do your parents encourage you to be PA** | 1.70 | 0.39 | 7.33 | 0.48 |
| **Duration of the school day** | 1.18 | 0.78 | 1.79 | 0.44 |
| **Transport for extracurricular activities** | 1.09 | 0.50 | 2.37 | 0.84 |
| **School healthy committee (yes)** | **0.22** | **0.10** | **0.49** | **0.00** |
| **Short breaks** *(one)* |  |  |  |  |
| *Two* | 2.61 | 0.97 | 7.06 | 0.06 |
| **Long breaks** *(one)* |  |  |  |  |
| *Two* | 1.85 | 0.70 | 4.84 | 0.21 |
| *Three* | 0.54 | 0.15 | 1.97 | 0.35 |
| **PE sessions per week** | **3.64** | **1.56** | **8.50** | **0.00** |
| **Duration PE (short)** |  |  |  |  |
| *Medium* | **11.70** | **1.55** | **88.09** | **0.02** |
| *Long* | **12.72** | **1.45** | **111.82** | **0.02** |
