# Supplementary material for: Prevalence and correlates of compliance with 24-h movement guidelines among children from urban and rural Kenya—The Kenya-LINX project
Source: PLoS One. 2022 Dec 30;17(12):e0279751. doi: 10.1371/journal.pone.0279751 (PMC9803245; doi:10.1371/journal.pone.0279751)
Supplement: Attachment — Submitted filename: Response to Reviewers.docx [file pone.0279751.s004.docx]

Dear Zulkarnain Jaafar,

On behalf of my co-authors, I would like to thank you for the opportunity to revise and resubmit our manuscript entitled **Prevalence and correlates of compliance with 24-h movement guidelines among children from urban and rural Kenya – The Kenya-LINX Project.** We have carefully considered the reviewers comments and adjusted the manuscript accordingly. On the following page, we have addressed each comment and provided a response in green. Corresponding changes are highlighted in the manuscript using track changes.

**Editor comments**

1. Please ensure that your manuscript meets PLOS ONE's style requirements, including those for file naming. The PLOS ONE style templates can be found at
   <https://journals.plos.org/plosone/s/file?id=wjVg/PLOSOne_formatting_sample_main_body.pdf> and 
   <https://journals.plos.org/plosone/s/file?id=ba62/PLOSOne_formatting_sample_title_authors_affiliations.pdf>.

Thank you for the guidance, the manuscript has been adjusted to follow the templates provided.

2. Please include a complete copy of PLOS’ questionnaire on inclusivity in global research in your revised manuscript. Our policy for research in this area aims to improve transparency in the reporting of research performed outside of researchers’ own country or community. The policy applies to researchers who have travelled to a different country to conduct research, research with Indigenous populations or their lands, and research on cultural artefacts. The questionnaire can also be requested at the journal’s discretion for any other submissions, even if these conditions are not met.  Please find more information on the policy and a link to download a blank copy of the questionnaire here: <https://journals.plos.org/plosone/s/best-practices-in-research-reporting>. Please upload a completed version of your questionnaire as Supporting Information when you resubmit your manuscript.

Questionnaire has been completed and attached.

3. In your Data Availability statement, you have not specified where the minimal data set underlying the results described in your manuscript can be found. PLOS defines a study's minimal data set as the underlying data used to reach the conclusions drawn in the manuscript and any additional data required to replicate the reported study findings in their entirety. All PLOS journals require that the minimal data set be made fully available. For more information about our data policy, please see <http://journals.plos.org/plosone/s/data-availability>. Upon re-submitting your revised manuscript, please upload your study’s minimal underlying data set as either Supporting Information files or to a stable, public repository and include the relevant URLs, DOIs, or accession numbers within your revised cover letter. For a list of acceptable repositories, please see <http://journals.plos.org/plosone/s/data-availability#loc-recommended-repositories>. Any potentially identifying patient information must be fully anonymized. Important: If there are ethical or legal restrictions to sharing your data publicly, please explain these restrictions in detail. Please see our guidelines for more information on what we consider unacceptable restrictions to publicly sharing data: <http://journals.plos.org/plosone/s/data-availability#loc-unacceptable-data-access-restrictions>. Note that it is not acceptable for the authors to be the sole named individuals responsible for ensuring data access. We will update your Data Availability statement to reflect the information you provide in your cover letter.

We are unable to make the data available on ethical grounds as ethical approvals and subsequently consent was obtained on the basis that data would only be shared with the research team. Specifically, the parent consent form contained the following statement “*The information you or your child provides will be kept private and the only people who will have access to the information are members of the research team*”.

*Revised data availability statement:*

Ethical approval for this study was granted by the Swansea University College of Engineering Research Ethics Committee and The Kenyatta University Ethical Review Committee, provided that participants’ data was only accessible by the research team. Data from this research study contains information at the individual and school level which when combined could be used to identify individuals. Participants of this study did not consent to having their data publicly available.

Requests to access the data may be directed to the Swansea University College of Engineering Research Ethics Committee [coe-researchethics@swansea.ac.uk](mailto:coe-researchethics@swansea.ac.uk) and the Ethics and scientific review committee at Kenyatta University [chairman.kuerc@ku.ac.ke](mailto:chairman.kuerc@ku.ac.ke), APPLICATION NUMBER PKU2106/11254

4. Please include captions for your Supporting Information files at the end of your manuscript, and update any in-text citations to match accordingly. Please see our Supporting Information guidelines for more information: <http://journals.plos.org/plosone/s/supporting-information>.

Thank you for pointing this out, captions have been included on page 29 lines 670-673.

5. Please review your reference list to ensure that it is complete and correct. If you have cited papers that have been retracted, please include the rationale for doing so in the manuscript text, or remove these references and replace them with relevant current references. Any changes to the reference list should be mentioned in the rebuttal letter that accompanies your revised manuscript. If you need to cite a retracted article, indicate the article’s retracted status in the References list and also include a citation and full reference for the retraction notice.

Thank you for this comment. We have checked each reference to ensure they are still available and relevant. To our knowledge none of the references have been retracted, however, the following changes have been made.

Page 24,

Reference 15, the name of the journal has been included in full.

Reference 16, the title has been edited to remove a typo.

Reference 30 capitalisation of title and the Doi have been corrected

Reference 50 – Correction made to the title

[Note: HTML markup is below. Please do not edit.]

Reviewers' comments:

Reviewer's Responses to Questions

**Comments to the Author**

1. Is the manuscript technically sound, and do the data support the conclusions?

The manuscript must describe a technically sound piece of scientific research with data that supports the conclusions. Experiments must have been conducted rigorously, with appropriate controls, replication, and sample sizes. The conclusions must be drawn appropriately based on the data presented.

Reviewer #1: Yes

Reviewer #2: Yes

2. Has the statistical analysis been performed appropriately and rigorously?

Reviewer #1: Yes

Reviewer #2: Yes

3. Have the authors made all data underlying the findings in their manuscript fully available?

The [PLOS Data policy](https://eur03.safelinks.protection.outlook.com/?url=http%3A%2F%2Fwww.plosone.org%2Fstatic%2Fpolicies.action%23sharing&data=05%7C01%7CN.J.Swindell%40Swansea.ac.uk%7Cf4c0b410ab4845bd1daa08dab5d7a863%7Cbbcab52e9fbe43d6a2f39f66c43df268%7C0%7C0%7C638022235220685417%7CUnknown%7CTWFpbGZsb3d8eyJWIjoiMC4wLjAwMDAiLCJQIjoiV2luMzIiLCJBTiI6Ik1haWwiLCJXVCI6Mn0%3D%7C3000%7C%7C%7C&sdata=YSbU6CBA%2FdEVidF0WKgceujgqxsJqaUtlM%2BrygV4ThU%3D&reserved=0) requires authors to make all data underlying the findings described in their manuscript fully available without restriction, with rare exception (please refer to the Data Availability Statement in the manuscript PDF file). The data should be provided as part of the manuscript or its supporting information, or deposited to a public repository. For example, in addition to summary statistics, the data points behind means, medians and variance measures should be available. If there are restrictions on publicly sharing data—e.g. participant privacy or use of data from a third party—those must be specified.

Reviewer #1: No

Reviewer #2: No

Thank you for taking the time to review our manuscipt and for the positive commenets.

As descibed above, we are not able to make the data available on ethical grounds as ethical approvals and subsequently consent was obtained on the basis that data would only be shared with the research team. The data availability statement has been updated to reflect the status of availability and offer contact information for the two university ethical committees.

4. Is the manuscript presented in an intelligible fashion and written in standard English?

PLOS ONE does not copyedit accepted manuscripts, so the language in submitted articles must be clear, correct, and unambiguous. Any typographical or grammatical errors should be corrected at revision, so please note any specific errors here.

Reviewer #1: Yes

Reviewer #2: Yes

5. Review Comments to the Author

Please use the space provided to explain your answers to the questions above. You may also include additional comments for the author, including concerns about dual publication, research ethics, or publication ethics. (Please upload your review as an attachment if it exceeds 20,000 characters)

**Reviewer #1:** As the title is the second published work so far in Sub-Saharan countries, it is well done. However, the format should follow PLOS ONE guideline. Hence, I suggest the authors to include limitation of the study, abbreviation, and authors contribution content.

Thank you for these suggestions. Study limitations can be found on page 21 lines 460 to 476. Author contributions have been added on page 29 line 671. Abbreviation have now all been described in full on first use (edit made line 110).

- Line 262, table 1, row 5: avoid such spacing which will affect the journal and the paper reputation
- Thank you for spotting this, spacing has not been adjusted.

  Spacing between headings are not uniform and blank page should be avoided.
  New paragraph should be double spaced from the other.
  Thank you for the comments, spacings have been adjusted throughout the document
- Corresponding author address should be full

Thank you for this comment, the details of the corresponding author have been adjusted to conform with the author guidelines (line 22) <https://journals.plos.org/plosone/s/file?id=ba62/PLOSOne_formatting_sample_title_authors_affiliations.pdf>. However, these guidelines did not recommend including address in full. If the address is required elsewhere in the submission, kindly advise.

**Reviewer #2**: This study examined compliance to the 24-hourmovement guidelines and their correlates among children from urban and rural Kenya. I would like to congratulate Authors for this interesting study. In general, the present study is worth publishing and adds valuable information. Nevertheless, I consider there are some changes that should be done.
Thank you for taking the time to review our manuscript and for the positive comments.

- Page 5, line 106: Please, explain an abbreviation of PE by providing its full form. Thank you for spotting this, this has been adjusted on line 110.
- Page 5, line 120: Please, give more detailed information about the participants – how did Authors recruit them (probability or convenience sampling)?

Thank you for this comment. The sampling framework used stratified random cluster sampling. Schools were randomly selected after being stratified by county (Nairobi/Kitui), type of school (private/public) and sub-county. As for the children, we used a full class participation approach of children with the eligible age and that had signed parental consent. We have included a line to describe this process (page 6 lines 130-132).

- Page 19, line 403 Several studies for me means more than two. Please add some references here.
- We agree with this comment and have edited the manuscript accordingly (page19, line 411)
